# Primary membranoproliferative glomerulonephritis in a child with down syndrome complicated with CVA: A case report

**DOI:** 10.1016/j.amsu.2022.104441

**Published:** 2022-08-18

**Authors:** Mohammad Khaled Alsultan, Zeina Nizar Bdeir, Ameer Obeid, Omar Alsamarrai, Hasan Nabil Al Houri, Qussai Hassan

**Affiliations:** a Department of Nephrology, Al Assad and Al Mouwasat University Hospitals, Damascus University, Faculty of Medicine, Damascus, Syria; b Department of Infectious Diseases, Al Assad and Al Mouwasat University Hospitals, Damascus University, Faculty of Medicine, Damascus, Syria; c Department of Neurology, Al Assad and Al Mouwasat University Hospitals, Damascus University, Faculty of Medicine, Damascus, Syria; d Department of Internal Medicine, Al Assad and Al Mouwasat University Hospitals, Damascus University, Faculty of Medicine, Damascus, Syria; e Faculty of Medicine, Al-Sham Private University, Damascus, Syria; f Department of Nephrology, Al Assad University Hospital, Damascus University, Faculty of Medicine, Damascus, Syria

**Keywords:** Membranoproliferative glomerulonephritis (MPGN), Down syndrome (DS), Cerebrovascular accident (CVA), Chronic kidney disease (CKD), Nephrotic syndrome (NS)

## Abstract

**Introduction:**

Down syndrome (DS) is a genetic disorder that affects multiple organs but glomerular lesions were reported only in case reports such as focal segmental glomerulosclerosis (FSGS), and Membranoproliferative glomerulonephritis (MPGN).

**Case presentation:**

A 14-year-old male child with DS was presented with generalized edema over three months. Laboratory tests revealed nephrotic syndrome (NS) and urinary tract infection (UTI). Renal ultrasound consisted with CKD. Kidney biopsy corresponded with MPGN. Also, all investigations for secondary underlying disorders came back negative suggesting the idiopathic form. Moreover, the status complicated with cerebrovascular accident (CVA), which has not been described in a DS- patient with glomerulonephritis.

**Discussion/conclusion:**

The relationship between DS and the incidence of glomerulonephritis is unclear. we suggest regular monitoring of renal function and urinalysis in different-age patients with Down syndrome, because early detection of renal disorders may prevent or slow down the progression and could be beneficial for increasing survival.

## Highlights

• Glomerulonephritis (GN) in the context of Down syndrome (DS) were only reported in case reports.
• A child with DS presented with nephrotic syndrome, CKD and the status complicated with CVA.
• Causal relationship between immunologic disorders in DS and the incidence of GN is unclear.
• This case supports the incidence of GN in different-age patients of DS.
• We suggest monitoring of renal function and urinalysis in patients of DS to early detect of renal disorders.

## 1 Introduction

Down syndrome (DS) is the main cause of chromosomal abnormality among newborns and the prevalence increased from 9.0 to 11.8 per 10 000 [1]. DS associates with a wide variety of abnormalities that is characterized by congenital heart anomalies, autoimmune disorders, hematologic disorders, and urinary tract anomalies (such as dysplastic kidney and renal hypoplasia) [2,3].

To our knowledge, Glomerular lesions in the context of DS were only reported in case reports, such as focal segmental glomerulosclerosis (FSGS) [2,4], IgA nephropathy, membranous glomerulonephritis, Pauci-immune crescentic GN, Lupus nephritis [2], ANCA-associated glomerulonephritis [5], and Membranoproliferative GN (MPGN) [2,6,7].

This report described a patient with down syndrome who presented with nephrotic syndrome and CKD. Renal histology revealing MPGN in the absence of an identifiable secondary disorder. This case report examines one such presentation in line with the SCARE guidelines [8].

## 2 Presentation of case

A 14-year-old male with DS was admitted to our nephrology department complaining with generalized edema for three-months, dyspnea, urinary burn and fever. The patient had history of recurrent urinary tract infection (UTI) and intellectual disability. There are no relevant family history of renal or other disorders. Physical examination revealed blood pressure 130/80, respiratory rate 24/min, heart rate 130 beats/min, temperature 38.5C, and oxygen saturation 96% on air room. He had severe lower extremities pitting edema, abdominal shifting dullness and diminished breathing sound in the right lung base.

Electrocardiogram (ECG) showed sinus tachycardia, and chest x-ray (CXR) demonstrated blunting of the right costophrenic angle. Echocardiography showed an ejection fraction (EF) of 55% with moderate pericardial effusion. Renal ultrasound showed small-size-kidneys (both kidneys measured 8 cm) with decreased cortical thickness and increased echogenicity, which consisted with CKD. Laboratory tests on admission show in Table 1, which consisted with glomerular disorder.

**Table 1:** Laboratory tests on admission.

Blood		HDL	20
WBC	23.5	TG	146
HB	7.1	CRP	2.3
PLT	374	ABGs	
Ur	167	PH	7.29
Cr	4.52	Bicarbonate	13
TP	5.6	Urinalysis	
ALB	2	color	turbid
Ca	6.8	PH	5
P	9.8	WBC (hpf)	100 c/μl
UA	8.2	RBCs (hpf)	1600 c/μl
Chol	128	protein	++
LDL	77	HB	+++

WBC; white blood count, HB; hemoglobin, PLT; platelets, Ur; urea, Cr; creatinine, TP; total protein, ALB; albumin, Ca; calcium, P; phosphorus, UA; uric acid, CRP; C-Reactive Protein (up to 0.5 mg/dl), Chol; cholesterol, LDL; low-density lipoprotein, HDL; high-density lipoprotein, TG; triglycerides, ABGs; arterial blood gas, HCO3; bicarbonate, hpf; per high-power field.

**Fig. 1:**
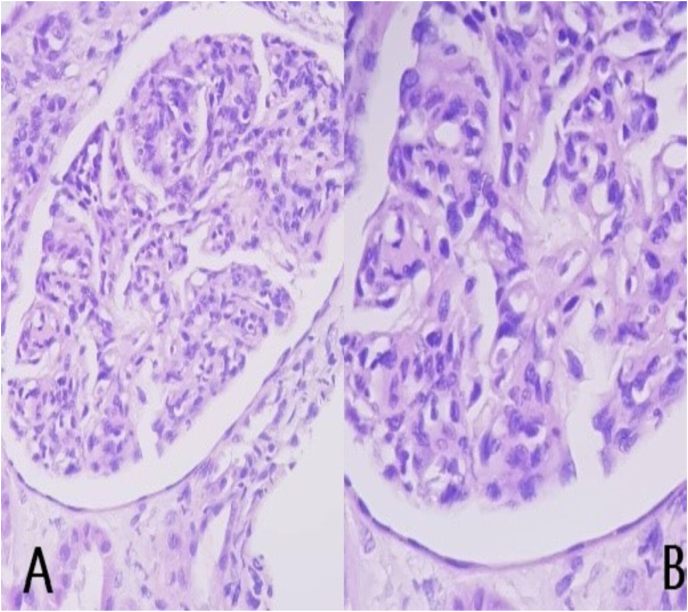
**PAS stain (A + B):** shows hypercellularity and lobulation of the glomerular tuft, thickening of glomerular basement membranes with mesangial expansion.

**Fig. 2:**
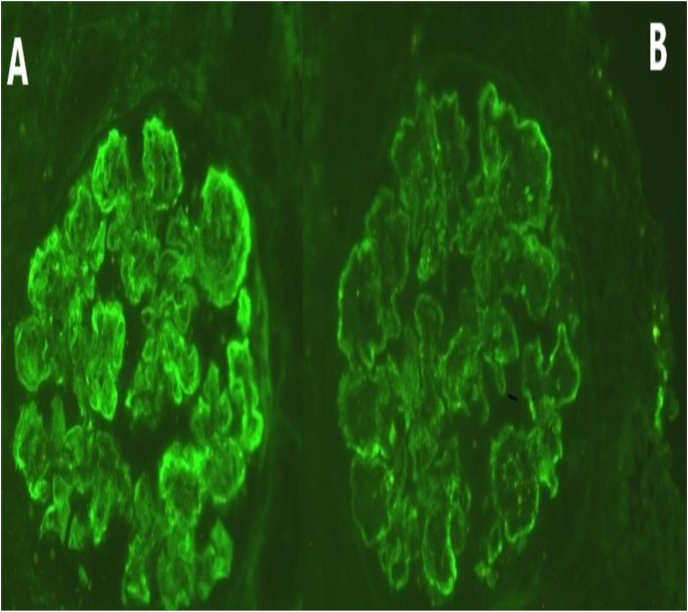
**Immunofluorescence (IF) (A + B):** shows diffuse segmental linear staining for IgG, C3, kappa, lambda and IgA.

Microscopically, the biopsy contained ten glomeruli and four were hyalinized. The glomerular architecture is distorted by hypercellularity and lobulation of the glomerular tufts. Glomerular basement membranes were thick and showed the presence of double contours by PAS stain (Fig. 1A). The capillary loops showed mild subintimal fibrosis and occasional hyaline droplet. The mesangial areas are expanded with PAS positive matrix and moderate to marked mesangial hypercellularity (up to 6 cells/mesangial area) (Fig. 1B). The interstitium showed scattered inflammatory cells. The masson stain showed interstitial fibrosis involving 25% of the cortex. Tubules showed focal atrophy and dilated lumens showing pus casts and hyaline casts admixed with red blood casts. The congo red stain was negative for amyloid. Immunofluorescence (IF) (Fig. 2 A + B) showed diffuse segmental linear staining for IgG (3+), C3, kappa and lambda (3+) and IgA (1+). Additional tests were also requested to exclude underlying disorders (Table 2) and all came back negative.

**Table 2:** Additional tests.

HBS Ag	Negative	SPEP	Low ALB
Anti HCV	Negative	24h urine test	
ANA	Negative	Volume	960 ml
Cryoglobulines	Negative	Cr	289 mg
C3	61	Protein	4300 ml
C4	16.6		

HBS Ag; hepatitis B surface antigen, Anti HCV; anti-hepatitis C antibodies, ANA; Antinuclear antibodies, C3; Complement C3 (80–160 mg/dl), C4; Complement C4 (15–45 mg/dl).

**Fig. 3:**
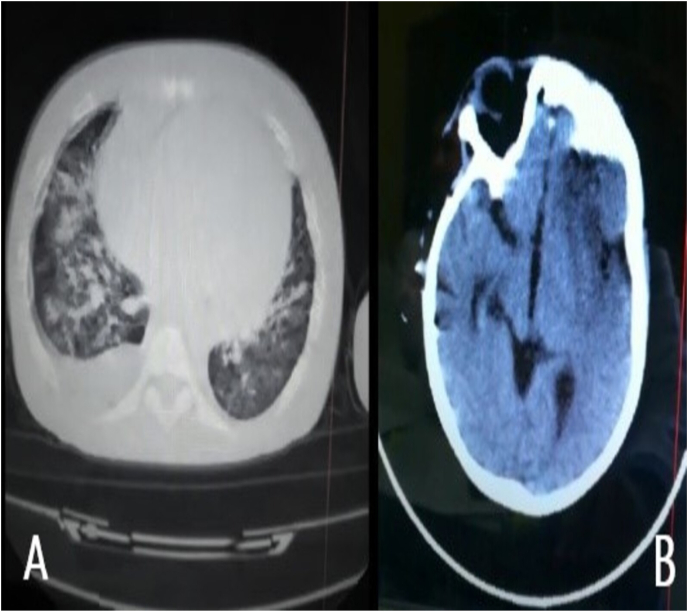
**CT scan** (A); chest CT shows bilateral infiltrations more dominant in the right lung with right pleural effusion, (B); brain CT shows decrease density on left frontal lobe compatible with ischemic stroke.

The patient was treated for urinary tract infection and discharged on high-dose prednisolone (40 mg/day) with subsequently tapering. Two weeks later he was readmitted with symptoms compatible with a respiratory infection. Chest CT revealed bilateral infiltrations and right pleural effusion, (Fig. 3A). The COVID-19 PCR was negative. A few days later the patient developed right hemiparesis and brain CT showed left frontal lobe stroke, (Fig. 3B). Thereafter, he was transmitted to ICU and dead after rapidly deterioration of severe respiratory distress.

## 3 Discussion

This report described a Down syndrome (DS) child who presented with NS, CKD and the status complicated with CVA. Pathologic manifestations correspond with idiopathic MPGN with global glomerulosclerosis.

To the best of our knowledge, only seven patients with MPGN and DS were reported in the literature. In a cohort study of 17 patients with DS, FSGS and IgA nephropathy were the most common forms of glomerular disorders. Only two patients were diagnosed with MPGN type I, one of them had hepatitis B infection. None of biopsies had tubulointerstitial or vascular disease and IF was negative for IgG in the both patients [2]. Whereas the patient in the current case had primary MPGN accompanied with 3+ IgG and fibrosis of the interstitium and capillary loops.

A previous study of four patients reported DS with MPGN type I. Three patients had proteinuria and low complement C3, moreover, only two patients had negative serologic tests for ANA, HBV and RF, the other two were not reported [6]. The present case showed similar results such as low C3 and proteinuria but all possible etiologies with serologic tests were excluded. One publication of DS reviewed two patients with renal failure and other 43 autopsies examinations, all had FSGS in addition to other types of glomerulonephritis but none of them were MPGN [4].

Generally, DS had a higher prevalence of autoimmune conditions [2]. Two cases of DS, the first reported ANCA-positive glomerulonephritis associated with diabetes mellitus, asthma and thyroiditis [5]. The second described three patients with a history of autoimmune disorders and only one patient had low complement MPGN [7]. In this report no previous immune disorders were found. Moreover, the coincidence of CVA and GN in a child with DS has not been described.

## 4 Conclusion

Here, a DS-patient was diagnosed with primary MPGN after exclusion of all secondary pathogenesis. Although a causal relationship between immunologic disorders in DS and the incidence of glomerulonephritis is unclear, our case along with previous reports supports the incidence of GN in different-age patients of DS. So, we suggest regular monitoring of renal function and urinalysis in patients with DS from early infancy to adulthood, because early detection of renal disorders may prevent or slow down the progression. Since, there were no large epidemiologic or observational studies to report the incidence or the relationship between GN and DS- patients, further studies were needed.

## Ethical approval

Written informed consent was obtained from the mother of the patient for publication of this case report and accompanying images, in line with local ethical approval requirements and in accordance with the helsinki declaration.

## Sources of funding

This research did not receive any specific grant from funding agencies in the public, commercial, or not-for-profit sectors.

## Author contribution

Dr. Mohammad Alsultan writes the manuscript, literature search and submit the article. Dr. Zeina Bdeir makes article corrections, literature search and patient follow up. Dr. Ameer Obeid makes article corrections, literature search and patient follow up. Dr. Omar Alsamurrai makes article corrections and writing a neurologic portion follow up. Dr. Hasan Al Houri makes article corrections.

## Trail registry number

1. Name of the registry: N\A.

2. Unique Identifying number or registration ID: N\A.

3. Hyperlink to your specific registration (must be publicly accessible and will be checked): N\A.

## Guarantor

The corresponding author is the guarantor of this manuscript.

## Consent

Written informed consent was obtained from the patient for publication of this case report and accompanying images. A copy of the written consent is available for review by the Editor-in-Chief of this journal on request.

## Provenance and peer review

Not commissioned, externally peer-reviewed.

## Declaration of competing interest

The author declares that they have no conflicts of interest regarding this study.

The author declares that it has not been published elsewhere and that it has not been submitted simultaneously for publication elsewhere.
